# A vital question—how to define inside and outside

**DOI:** 10.1007/s00709-025-02120-7

**Published:** 2025-10-06

**Authors:** Peter Nick

**Affiliations:** Joseph Gottlieb Kölreuter Institute Für Plant Sciences, Karlsruhe Institute of Technology, Karlsruhe, Germany

Life moves along the borderlines of physics—for instance, by inverting entropy in one site, at the cost of increased entropy elsewhere. This works only by separating spaces differing in their chemistry. These borders must be at the same time permeable and impermeable, depending on the chemical nature of the molecules that encounter this border. On the cellular level, this function is conveyed by bio-membranes endowed with a distinct set of pores and transporters. On the organismic level, often specific tissues adopt the role of a living barrier. The physicochemical constraints deriving from the second law of thermodynamics imply that life must be cellular; there is nothing like a *vis vitalis* that can travel through space and incarnate in inanimate matter to create a living organism. As suggested by the current theories on its origin, life started in compartments delineated by a semi-permeable sheath from pyrite inserted into chemical gradients generated by geochemical phenomena (for review, see Martin and Russell 2003). Four contributions to the current issue deal with the question of how, in plants or in animals, the inside is delineated against a challenging outside.

This delineation can transcend a merely physical barrier, as shown in the contribution by Wang et al. (2025) dealing with the intestinal barrier in pigs. The intestinal barrier represents a complex of physical (tight junctions in the epithelium), chemical (mucosic layers for protection), immunity-related (cytokines and immune cells in the *lamina propria*), and ecological (beneficial microbes) components. The motivation for this study derives from the particular challenges of high altitude in the Tibetan plateau, mainly the high exposure to damaging UV radiation, rendering animal husbandry difficult. The authors test the potential of a particular species of Wolfberry, *Lycium ruthenicum*, used in Traditional Chinese Medicine, as a functional food supplement for pig feeding. This berry, known in China under the designation *ninxia goji*, accumulates high levels of different anthocyanins and, therefore, appears black. It can cope even with high altitudes, probably linked with a strong antioxidant activity. The authors demonstrate that this berry improves the intestinal barrier on several levels, such as downmodulating inflammation responses controlled by the Toll-interleukin pathway, stimulating the expression of antioxidant enzymes, proteins involved in the establishment of tight junctions, as well as in mucus production, and improving the gut microbiome. Thus, they find significant improvements in all components of the intestinal barrier. While it is not possible, at this stage, to define primary and secondary changes because the phenomena are interrelated, the study illustrates convincingly the potential of this traditional nutraceutical and also represents a good example of hypothesis-driven application.

Also, the contribution by Sırrı and Mutlu (2025) is addressing biological barriers against a hostile environment, this time in a phytophagous desert beetle, *Cassida palaestina*, found in arid areas of the Middle East. The authors investigate the Malpighian tubules by classic anatomy and scanning electron microscopy. These analogues of the kidney form as appendices of the midgut, but are separated by a so-called peritrophic membrane, which allows actively importing potassium and sodium ions from the hemolymph. The ion gradient drives the import of water and ureic acid. The latter crystallises and is thus removed from chemical equilibrium sustaining water uptake. The authors observe that the distal end of the tubules contacts the hindgut, a condition termed as cryptonephridial and considered as an adaptation to arid environments, because it is missing in water-living beetles. The presence of microvilli and granules in the cytoplasm of epithelial cells is an indicator of strong physiological activity, reflecting the adaptation of this species to water scarcity. The anatomical details of this barrier can explain the ecological success of this type of beetles that perform well under conditions of climate change, as shown by recent modeling (Sırrı and Bal 2025).

Transport of sodium and potassium is not only crucial for the adaptation of insects to arid environments, but also pivotal for crop performance under the conditions of climate change. The conditions can be even harsher when drought is accompanied by salinity, which is true for many agricultural areas in Tunisia. Traditionally, resilient cereals such as barley dominated. Especially autochthonous varieties are often endowed with significant salt tolerance, while modern high-yielding cultivars often perform poorly under salinity. The study by Fathalli et al. (2025) investigates the mechanisms behind this phenomenon. Comparing salt-tolerant landraces from Tunisia with a current elite cultivar, they can link salt tolerance with a reduced transfer of sodium from the root to the shoot. Sodium accumulation comes with a depletion of potassium, and indeed, the tolerant variety can sustain higher levels of potassium. The authors search for the underlying mechanism and focus on high-affinity potassium transporters and report that these transporters are upregulated in the roots of the tolerant variety. Since sodium can move in the intercellular space of the root cortex, it is mainly the membrane of the endodermis where plants can prevent harmful ions from entering the plant body. In this tissue layer, the intercellulars are sealed off by the impermeable Casparian strip, such that the water with the solvent has to pass a membrane barrier. The high-affinity potassium transporters can ensure at this membrane that more potassium can enter the central cylinder, which means simultaneously a discrimination against sodium. Again, the homeostasis of the inner space is defended against external challenges using a combination of physical (the Casparian strip) and physiological (upregulated high-affinity potassium uptake transporters) mechanisms.

The contribution by Tarasenko et al. (2025) to the current issue carries the question of barriers between inside and outside into the cells. Eukaryotic cells derive from endosymbiotic events, where prokaryotic cells were ingested and later domesticated by gene transfer. Since many proteins are encoded in the nucleus and need to be imported into the organelle, the expression of nuclear genes needs to be synchronised with that in the remnant organelle genome. Organelle import of proteins has been intensively studied, but in addition to proteins, nucleic acids can also cross the organellar boundary. This not only includes some of the tRNAs, but also short DNA fragments. The authors studied the underlying molecular players and show that the Tric1/Tric2 proteins (for tRNA import component) normally responsible for tRNA import and Tom20-2, a subunit of the protein translocase, are required to import such DNA fragments, up to around 250 bp, into the stroma of Arabidopsis plastids, complementing a previously characterised uptake system for long DNA involving the porin VDAC1. The Tric proteins can dimerise, both with themselves and with other partners, which allows generating channels with different sizes and cargo affinities. Thus, the components of the machinery that selectively transport either proteins, tRNA, or DNA across the membrane of plant mitochondria are shared, and the specificity seems to be generated by modular combinations of these components.

The four different contributions illustrate the versatility and complexity by which the boundaries between inside and outside are established. These boundaries are as well connecting as they are separating. While physical separation, either by lipid membranes (on the cellular level) or tissues (on the organismic level), seems to be a commonality of biological barriers, it is complemented and sustained by biological processes. These processes can be physiological, as in the case of the intestinal barrier involving immunity and commensalistic microbes. They can also be biochemical, as for the high-affinity potassium transporters in barley or in the case of the mitochondrial membrane, where cargo specificity of transporters is achieved by modular combination. Thus, it might be fruitful to conceive biological barriers not as static borders but rather as activity creating and sustaining the autonomy of an internal space against the adversities of a fluctuating and hostile environment.
